# A genome‐wide CRISPR screen identifies *FBXO42* involvement in resistance toward MEK inhibition in *NRAS*‐mutant melanoma

**DOI:** 10.1111/pcmr.12825

**Published:** 2019-10-10

**Authors:** Adi Nagler, David W. Vredevoogd, Michal Alon, Phil F. Cheng, Sophie Trabish, Shelly Kalaora, Rand Arafeh, Victoria Goldin, Mitchell P. Levesque, Daniel S. Peeper, Yardena Samuels

**Affiliations:** ^1^ Department of Molecular Cell Biology Weizmann Institute of Science Rehovot Israel; ^2^ Division of Molecular Oncology and Immunology The Netherlands Cancer Institute Amsterdam The Netherlands; ^3^ Department of Dermatology University of Zurich Hospital Zurich Switzerland

**Keywords:** CRISPR, melanoma, NRAS, resistance, therapy

## Abstract

*NRAS* mutations are the most common alterations among RAS isoforms in cutaneous melanoma, with patients harboring these aggressive tumors having a poor prognosis and low survival rate. The main line of treatment for these patients is MAPK pathway‐targeted therapies, such as MEK inhibitors, but, unfortunately, the response to these inhibitors is variable due to tumor resistance. Identifying genetic modifiers involved in resistance toward MEK‐targeted therapy may assist in the development of new therapeutic strategies, enhancing treatment response and patient survival. Our whole‐genome CRISPR‐Cas9 knockout screen identified the target Kelch domain‐containing F‐Box protein 42 (FBXO42) as a factor involved in *NRAS*‐mutant melanoma‐acquired resistance to the MEK1/2 inhibitor trametinib. We further show that FBXO42, an E3 ubiquitin ligase, is involved in the TAK1 signaling pathway, possibly prompting an increase in active P38. In addition, we demonstrate that combining trametinib with the TAK1 inhibitor, takinib, is a far more efficient treatment than trametinib alone in *NRAS*‐mutant melanoma cells. Our findings thus show a new pathway involved in *NRAS*‐mutant melanoma resistance and provide new opportunities for novel therapeutic options.

**Significance**

As *NRAS*‐mutant melanoma tumors are mostly resistant to MEK inhibitors, investigating signaling pathways that lead to resistance has taken a center stage. Our data show that the TAK1 pathway is involved in resistance toward MEK inhibition in *NRAS*‐mutant melanoma. These findings have clinical implications as they may lead to the development of combined inhibitor therapy toward MEK and TAK1, which could be an effective treatment for melanoma patients harboring an *NRAS* mutation.

## 1 INTRODUCTION

*NRAS* mutations are found in 15%–20% of melanomas (Hodis et al., 2012). Tumors harboring these mutations are very aggressive, displaying elevated mitotic activity and high rates of lymph node metastasis (Devitt et al., 2011; Jakob et al., 2012). Thus, patients with *NRAS*‐mutant melanoma have a poor prognosis and a low survival rate (Jakob et al., 2012; Thumar, Shahbazian, Aziz, Jilaveanu, & Kluger, 2014).

Despite decades of research on RAS, it is still regarded as being “undruggable” (Stephen, Esposito, Bagni, & McCormick, 2014). Therefore, MAPK pathway inhibitors, such as MEK inhibitors (MEKi), are the typical therapeutic approach when it comes to *NRAS*‐mutant melanoma (Munoz‐Couselo, Adelantado, Ortiz, Garcia, & Perez‐Garcia, 2017; Santarpia, Lippman, & El‐Naggar, 2012). Trametinib is an FDA‐approved, allosteric inhibitor of MEK1/2 used to treat *NRAS*‐mutant melanoma, both as a monotherapy and in combination with other anti‐cancer drugs (Johnson & Puzanov, 2015). However, these current therapies used to treat patients with *NRAS*‐mutant melanoma are not very efficient, owing to the aggressive nature of tumor cells and complex changes in molecular signaling (Johnson & Puzanov, 2015). Thus, identifying genetic modifiers involved in such resistance mechanisms is of great importance. We here report on a novel mechanism of drug resistance in *NRAS*‐mutant melanoma.

Our whole‐genome CRISPR‐Cas9 knockout (KO) screen in *NRAS^Q61R^* melanoma cells revealed the Kelch domain‐containing F‐Box protein 42 (FBXO42), an E3 ubiquitin ligase (Sun et al., 2009), involvement in resistance toward trametinib treatment. We further show that FBXO42 is involved in the TAK1 signaling pathway, leading to increased P38 activation. Thus, based on these observations, we demonstrate that combining trametinib with takinib, a TAK1 inhibitor is a far more efficient treatment than monotreatment with trametinib in *NRAS*‐mutant melanoma cells.

Put together, our findings reveal a novel mechanism of tumor resistance to MEK inhibition in *NRAS*‐mutant melanoma, and potentially offering a new therapeutic strategy.

## 2 MATERIALS AND METHODS

### 2.1 Cell culture

SK‐MEL‐147 (NRAS Q61R) and MZ‐MEL‐2 (NRAS Q61K) cell lines were a gift from Prof. Daniel S. Peeper (The Netherlands Cancer Institute, Amsterdam, The Netherlands). MM130405, MM130926, and MM130227 (NRAS Q61R) cell lines were a gift from Prof. Mitch Levesque (University of Zurich Hospital, Zurich, Switzerland). SK‐MEL‐147 and MZ‐MEL‐2 cell lines were cultured in DMEM. MM130405, MM130926, and MM130227 cell lines were cultured in RPMI‐1640. All melanoma cell lines were supplemented with 10% FBS, L‐glutamine, penicillin, and streptomycin and grown at 37°C in 5% CO_2_ for 5–15 passages. All cells have been authenticated by sequencing and were tested routinely for mycoplasma using Mycoplasma EZ‐PCR test kit (#20‐ 700–20, Biological Industries, Kibbutz Beit Ha'emek).

### 2.2 Genome‐wide CRISPR‐Cas9 knockout (GeCKO) screen

#### 2.2.1 Lentivirus production of GeCKO library

Lentivirus was produced as previously described (Shalem et al., 2014). Briefly, 4.4 µg of the lentiCRISPRv2 library (#1000000048, Addgene) was co‐transfected with 2.2 µg of PMD2.G (#12259, Addgene), and 3.4 µg of psPAX2 (#12260, Addgene) packaging plasmids into HEK293T cells in a 10 cm^2^ dish using TurboFect (Thermo Fisher) as described by the manufacturer. After 60 hr, media were collected, filtered, aliquoted, and stored at −80°C.

#### 2.2.2 CRISPR‐Cas9 mediated genome‐wide screen

SK‐MEL‐147 cells were lentivirally transduced with two GeCKO libraries (A and B) at a MOI of 0.3 aiming to ensure that most cells receive only one viral construct (Shalem et al., 2014). Briefly, 5 × 10^6^ cells were plated in 10 cm^2^ dishes. 48 hr after infection, cells were selected with puromycin (1 µg/ml) for 14 days. Cells were split into two pools: One arm was subjected to 100 nM MEKi trametinib treatment (GSK1120210 Selleckchem), whereas the other arm was left untreated. Colonies formed in the drug‐treated arm were individually picked and expanded. For identification of sgRNAs in the individual clones, genomic DNA was isolated and sgRNAs were recovered by PCR amplification. Amplified DNA fragments were cloned into the TOPO TA‐cloning vector (450071, Invitrogen), followed by identification of the sgRNA by Sanger sequencing.

### 2.3 CRISPR cloning

Cloning of sgRNAs into the LentiCRISPRv2 vector was performed as described (http://www.genome-engineering.org/crispr/). Briefly, the LentiCRISPRv2 plasmid was digested with BsmBI and gel‐purified. DNA oligonucleotides (Invitrogen) were annealed and ligated into the digested vector. Target sgRNA oligonucleotide sequences are listed in Figure S8.

### 2.4 Western blot analysis

Cells were gently washed two times in PBS and then lysed in sample buffer 2X. The extracts were sonicated (50 W, 30 s), incubated on ice for 15 min, and boiled for 5 min. The samples were then subjected to 10% SDS‐PAGE. Immunoblots were probed with the following antibodies: pERK1/2 (4370S;1:1000), ERK1/2 (4695;1:1000), P38 (9212S; 1:1000), pP38 (9216S; 1:1000), JNK (9258;1:1000), and pJNK (9251S;1:1000) antibodies were obtained from Cell Signaling. GAPDH (MAB374;1:1000), Tubulin (05–829;1:1000), RAS, and RAS‐GTP (RAS activation kit) antibodies were obtained from Millipore. FBXO42 (ab81638;1:500) was obtained from Abcam.

Blots were developed with HRP‐conjugated anti‐mouse or anti‐rabbit Abs, using SuperSignal West Pico Chemiluminescent Substrate or SuperSignal West Femto Chemiluminescent Substrate from Thermo Scientific. pERK trametinib‐treated Western blots were developed using SuperSignal West Femto Chemiluminescent. Pictures of the blots were taken using Bio‐Rad ChemiDoc MP System. Quantification was done using Image Lab (Bio‐Rad).

### 2.5 Colony formation assay

Colony formation assays were performed by seeding 500 K cells in 6‐well plates. The medium was refreshed twice per week for 2 weeks, and then, the plates were fixed in 4% formaldehyde solution, stained with crystal violet (0.01% in dH2O), and photographed.

### 2.6 Cell viability assays

Melanoma cell lines were seeded into 96‐well plates (3,000 cells per well). On the next day, trametinib (GSK1120210 Selleckchem)/ selumetinib (AZD6244, AstraZeneca) was added to the plate's wells at increasing concentrations, from 1 pM to 100 μM, in three replicates, with DMSO as a negative control. After an additional 72 hr, cell proliferation was assessed using the Cell Titer‐Glo Luminescent Cell Viability Assay (Promega). Analysis was performed using graphPad Prism. The combined effect of TAK1 and MEK inhibition on cell proliferation was tested by adding to the plate wells an increasing concentration of the MEK inhibitor trametinib, from 1 pM to 100 μM, and a constant concentration of 5 μM takinib (HY‐103490, BioTAG). Cells were evaluated for viability after 72 hr as described above.

### 2.7 RNA sequencing analysis

RNA capture was performed with TruSeq Library Prep Kit v2 (Illumina) and sequenced on a HiSeq4000. RNA counts were quantified from single‐end reads using STAR aligner (Dobin et al., 2013). Differential expression was performed using voom from R package limma (Law, Chen, Shi, & Smyth, 2014).

### 2.8 Immunohistochemistry

Tissue sections (4 μm thick) were deparaffinized in xylene, rehydrated using graded concentrations of ethanol, and rinsed in distilled water. Heat‐induced epitope retrieval was performed in 10 mM citrate buffer at pH 6.0 for 10 min at 95°C. Sections were allowed to cool for one hour and then rinsed in distilled water. Endogenous peroxidase activity was blocked for 30 min. with hydrogen peroxide. For nonspecific binding, sections were blocked with 20% normal horse serum and 0.1% triton. Following blocking treatment, primary antibody (Rabbit anti‐human FBXO42 obtained from Abcam [ab81638]) was diluted 1:25 and incubated overnight at 4°C. Detection was accomplished using a biotinylated secondary goat anti‐rabbit antibody, followed by application of streptavidin–peroxidase conjugate solution and exposure to 3– 3′‐diamino‐benzidine (Sigma). Slides then were counterstained with hematoxylin (Sigma), dehydrated, and mounted with permanent media. Stained sections were examined and photographed on a bright‐field scanner (Pannoramic SCAN II slide scanner) equipped with Carl Zeiss objectives (10×; 20×; 40×; 60×).

### 2.9 Pooled stable expression

To produce lentiviruses, the following FBXO42 constructs were generated: FBXO42 was tagged with Flag at the N‐terminus (pCDH1‐FBXO42), FBXO42Δfbox was tagged with Flag at the C‐terminus, and FBXO42Δkelch was tagged with Flag at the N‐terminus. Deletion mutations were a kind gift from Yongfeng Shang (Peking University Health Science Center). Plasmids were co‐transfected into HEK293T cells seeded at 2.5 × 10^6^ per T75 flask with psPAX2 and pMD2.G helper plasmids using TurboFect as described by the manufacturer. Virus‐containing media were harvested 60 hr after transfection, filtered, aliquoted, and stored at −80°C. The lentiviruses for FBXO42 and its mutants were used to infect SK‐MEL‐147, MM130926, and MM130227 as previously described (Arafeh et al., 2015).

### 2.10 RAS activation assay

Two 15‐cm plates with SK‐MEL‐147 melanoma cells were treated with 100 nM trametinib or DMSO as control, for 24 hr. Ras‐GTP levels were detected using a Ras activation kit (Millipore), following the manufacturer's instructions (Merck). RAS‐GTP activation was quantified by using Image Lab software (Bio‐Rad).

### 2.11 Immunoprecipitation

SK‐MEL‐147 cells stably expressing wild‐type or mutant FBXO42 or empty vector were gently washed two times in PBS, trypsinized, and then lysed using a lysis buffer (1% NP‐40, 50 mM Tris‐HCl pH 7.5, 150 mM NaCl, 0.5% deoxycholic acid, 1% complete protease inhibitor (Roche), 1 mM sodium orthovanadate, 1 mM sodium fluoride, and 0.1% SDS in DDW). Lysates were incubated for 15 min on ice and then centrifuged for 15 min at 16,000 *g* at 4°C. 3 mg of the protein lysates was taken for immunoprecipitation using anti‐Flag M2 agarose beads (Sigma) in rotation overnight at 4°C. Proteins (50 μg/lane) were resolved by 10% SDS‐PAGE and transferred to nitrocellulose membranes (Bio‐Rad). Immunoblots were probed with the following antibodies: anti‐FLAG (M2) (F7425, Sigma) and anti‐GAPDH (MAB374, Millipore).

### 2.12 Identification of FBXO42‐interacting proteins

Immunoprecipitation of FBXO42 in SK‐MEL‐147 cells was performed as previously described.

Immunoprecipitations were washed five times with lysis buffer and then resuspended with sample buffer before denaturation and separation by SDS‐PAGE on 10% mini gels.

The proteins in the gel were visualized with an Imperial™ protein stain (Thermo Scientific), then reduced with 3 mM DTT (60°C for 30 min), modified with 10 Mm iodoacetamide in 100 mM ammonium bicarbonate (in the dark, room temperature for 30 min), and digested in 10% acetonitrile and 10 mM ammonium bicarbonate with either modified trypsin or chymotrypsin (Promega) at a 1:10 enzyme‐to‐substrate ratio, overnight at 37°C. Alternatively, the proteins in a mixture in 8 M urea and 100 mM ammonium bicarbonate were reduced and modified as described and digested in 2 M urea, 25 mM ammonium bicarbonate with modified trypsin or chymotrypsin (Promega) at a 1:50 enzyme‐to‐substrate ratio. The resultant peptides were desalted using C18 tips (Homemade stage tips) and subjected to LC‐MS‐MS analysis. The peptides were resolved by reverse‐phase chromatography on 0.075 × 180‐mm fused silica capillaries (J&W) packed with Reprosil reversed phase material (Dr. Maisch GmbH). The peptides were eluted with a linear 30‐min gradient of 5%–35% acetonitrile with 0.1% formic acid in water, 15‐min gradient of 35%–95% acetonitrile with 0.1% formic acid in water, and 15 min at 95% acetonitrile with 0.1% formic acid in water at a flow rate of 0.15 μl/min. Mass spectrometry was performed with a Q Exactive plus mass spectrometer (Thermo) in a positive mode using repetitively full MS scan, followed by high‐energy collision‐induced dissociation (HCD) of the 10 most dominant ions selected from the first MS scan. The mass spectrometry data were analyzed using Proteome Discoverer 1.4 software with Sequest (Thermo) and Mascot (Matrix Science) algorithms against a human UniProt database with a mass tolerance of 10 ppm for the precursor masses and 0.05 amu for the fragment ions. Oxidation on Met was accepted as a variable modification, and carbamidomethyl on Cys was accepted as a static modification. The minimal peptide length was set to six amino acids, and a maximum of two miscleavages was allowed. Peptide‐ and protein‐level false discovery rates (FDRs) were filtered to 1% using the target‐decoy strategy. Semi‐quantitation was done by calculating the peak area of each peptide based on its extracted ion currents (XICs), and the area of the protein was determined by averaging the three most intense peptides from each protein.

## 3 RESULTS

### 3.1 A function‐based genomic screen in *NRAS*‐mutant melanoma cells identifies *FBXO42* loss driving trametinib resistance

**Figure 1:**
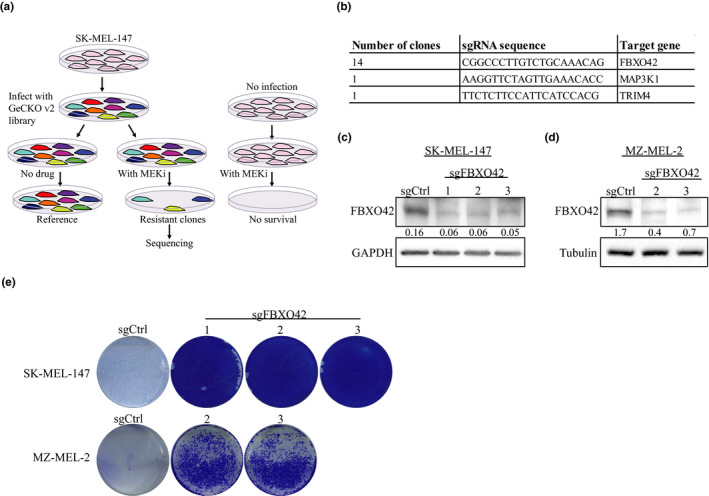
Genome‐wide CRISPR Cas9 knockout screen identifies FBXO42 leading to trametinib resistance in *NRAS*‐mutant melanoma. (a, b) Screen outline and hits, FBXO42 was found in 14 independent clones, and MAP3K1 was found in one clone out of the 14 as was TRIM4. (c,d) Immunoblot analysis of *FBXO42* gene perturbation efficiency of Cas9 sgRNAs in SK‐MEL‐147 and MZ‐MEL‐2 *NRAS*‐mutant melanoma cell lines. Ratios of FBXO42 to GAPDH and tubulin levels were generated using Image Lab (Bio‐Rad). (e) SK‐MEL‐147 and MZ‐MEL‐2 cells were treated with 100 nM trametinib and stained with crystal violet 10 days later

In order to identify genes essential to maintain sensitivity toward the MEKi trametinib in *NRAS*‐mutant melanoma, we conducted a whole‐genome CRISPR‐Cas9 knockout screen **(**Figure 1a).

We chose to use SK‐MEL‐147, a commonly used melanoma cell line harboring the recurrent *NRAS^Q61R^* mutation and highly sensitive to trametinib treatment. SK‐MEL‐147 cells were transduced with the human GeCKO (Shalem et al., 2014) v2 library. Cells were selected for stable viral integration with puromycin for 14 days. Next, the cells were split into two pools: One arm was treated with trametinib, whereas the other was left untreated as a control. Thirty days post‐drug treatment, 14 trametinib‐resistant colonies emerged and were sequenced. All the colonies contained the same single‐guide RNA (sgRNA) targeting the gene *FBXO42* (Figure 1b). Two of the 14 resistant colonies contained additional sgRNAs targeting *MAP3K1* and *TRIM4*. We focused on *FBXO42* since it was identified in all resistant colonies.

**Figure 2:**
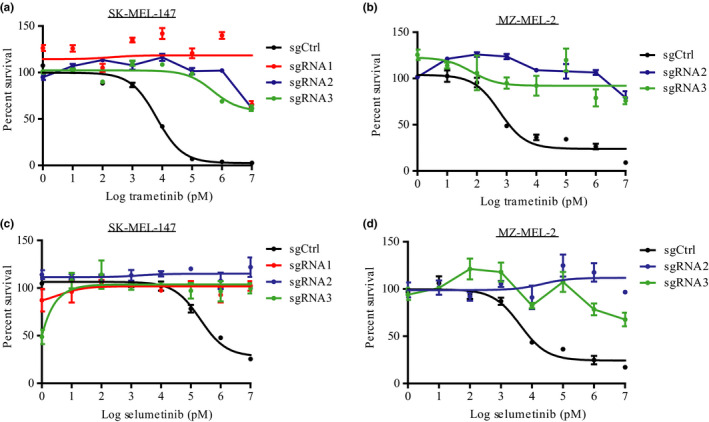
*FBXO42* KO leads to MEKi resistance in *NRAS* mutant cell lines. (a‐d) Dose–response curves generated using SK‐MEL‐147 and MZ‐MEL‐2 cell lines treated with trametinib or selumetinib (1 pM–10 μM) for 72 hr before assessing cell viability using Cell Titer‐Glo Luminescent Cell Viability Assay (*n* = 3). The relative cell number post‐trametinib treatment is plotted as percent survival versus log trametinib concentration in pM. Error bars, *SD*

To determine whether *FBXO42* KO indeed leads to resistance in *NRAS*‐mutant melanoma cells, we knocked out *FBXO42* from *NRAS* mutant cell lines. We used SK‐MEL‐147 cell line as used in the CRISPR screen, the commonly used melanoma cell line MZ‐MEL‐2 and patient‐derived cell line MM130405 (Figure 1c,d and Figure S1a, S8). Next, we added trametinib treatment and performed colony formation assays. The *FBXO42* KO cells resulted in a significant increase in the colony number compared to control non‐targeting sgRNA cells (Figure 1e and Figure S1b). In addition, we tested the effect of *FBXO42* KO on the cells' viability using cell titer‐glo assay. We showed an increase in cell viability in *FBXO42* KO samples treated with trametinib, compared to the control (Figure 2a,b and Figure S1c). We checked the resistance effect of these cell lines toward an additional potent and highly selective MEK1/2 inhibitor, selumetinib, (Kim & Patel, 2014) and received similar results (Figure 2c,d). These data confirm that KO of *FBXO42* in *NRAS*‐mutant melanoma cells leads to resistance toward trametinib treatment.

### 3.2 FBXO42 is a predictive biomarker of trametinib resistance in *NRAS*‐mutant melanoma

**Figure 3:**
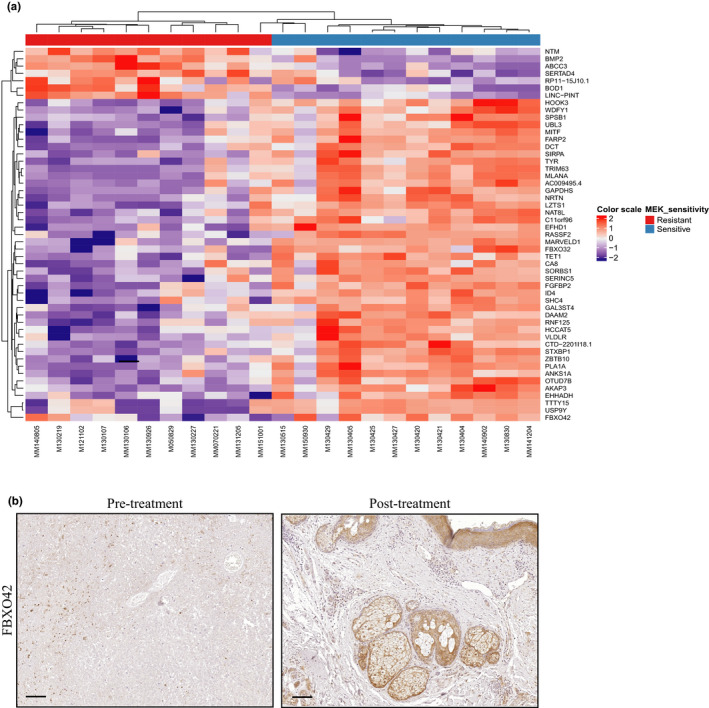
*FBXO42* is a predictive biomarker leading to trametinib resistance in *NRAS*‐mutant melanoma. (a) RNA sequencing performed on 23 *NRAS* mutant cell lines derived from melanoma patients treated with MEKi. Top bar indicates MEKi‐resistant patients in red, MEKi‐sensitive patients in blue. Scale bar indicates the expression level of the genes in the Y axis. (b) Representative immunohistochemical stain for FBXO42 in melanoma tumor slides taken from patients sensitive to MEKi treatment. Image is presented in ×10 magnification, scale 100 μm

**Figure 4:**
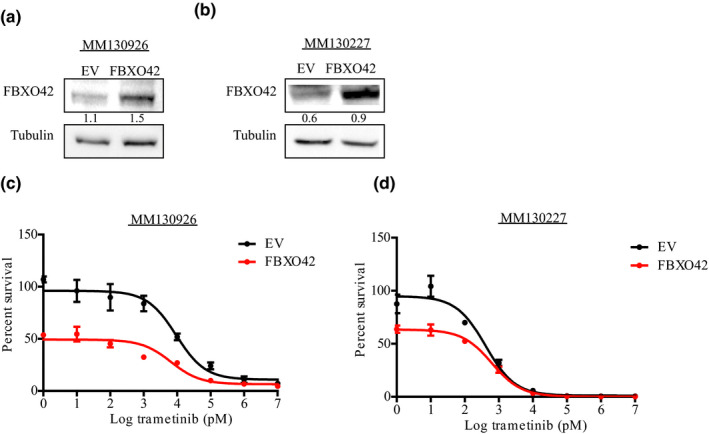
*FBXO42* overexpression sensitizes *NRAS*‐mutant melanoma cell lines to trametinib treatment. (a,b) Immunoblot analysis of *FBXO42* gene overexpression efficiency in MM130926 and MM130227 patient‐derived *NRAS*‐mutant melanoma cell lines. Ratios of FBXO42 to tubulin levels were generated using Image Lab (Bio‐Rad). (c,d) Dose–response curves generated using MM130926 and MM130227 cell lines treated with trametinib (1 pM–10 μM) for 72 hr before assessing cell viability using Cell Titer‐Glo Luminescent Cell Viability Assay (*n* = 3). The relative cell number post‐trametinib treatment is plotted as percent survival versus log trametinib concentration in pM. Error bars, *SD*

*FBXO42* was differentially expressed between 23 resistant and sensitive patients treated with MEK inhibitors. Out of the 12 cell lines derived from patients sensitive to MEKi, eight show increased expression of *FBXO42* (Figure 3a). Complementary, immunohistochemistry staining of patient samples before and after MEKi treatment shows elevated *FBXO42* expression in patient sensitive to the treatment (Figure 3b**)**. Overexpression of *FBXO42* in the MEKi‐resistant patient‐derived cell lines, MM130926 and MM130227, showed a decrease in cell viability compared to control (Figure 4). This implies that FBXO42 may play a role in upfront resistance in *NRAS*‐mutant melanoma.

### 3.3 *FBXO42* KO leads to MAPK pathway activation

**Figure 5:**
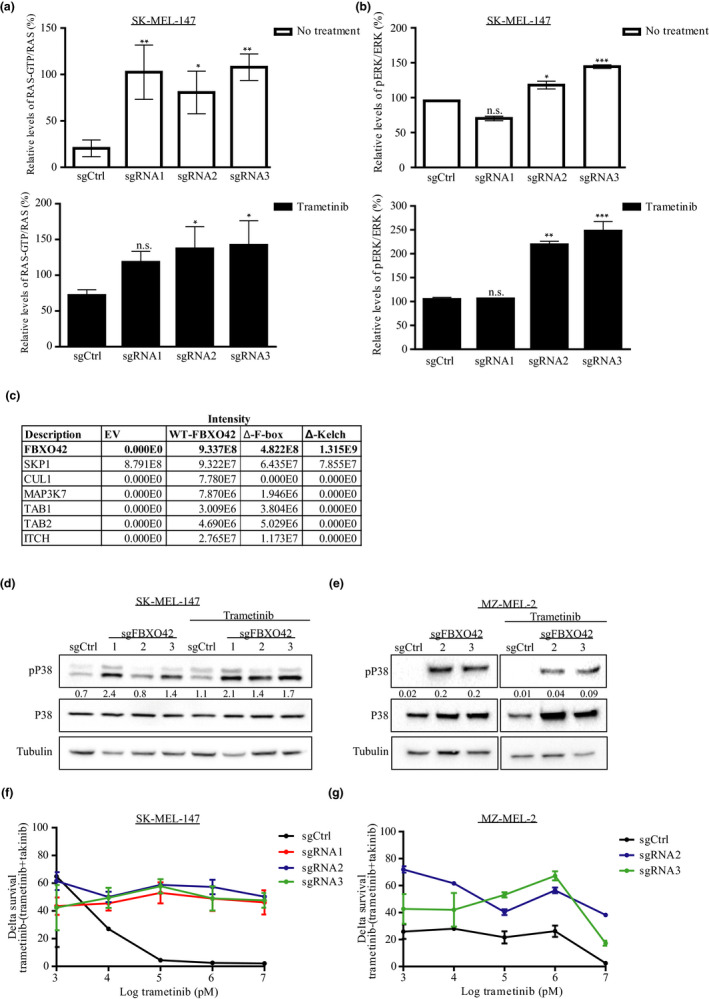
*FBXO42* KO activates the MAPK pathway and TAK1 signaling leading to trametinib resistance. (a) SK‐MEL‐147 RAS‐GTP levels were assessed by RAS pull‐down assay, and treated samples were incubated with 100 nM trametinib for 24 hr. RAS‐GTP/RAS ratios are representative of three independent experiments. Trametinib‐treated and non‐treated samples were compared to their sgCtrl. *n* = 3, *p < .05, **p < .01 one‐way ANOVA followed by Tukey's test. (b) SK‐MEL‐147 cells were treated with 100 nM trametinib for 24 hr. Cell lysates were analyzed by immunoblot. pERK/ERK ratios were calculated from two independent experiments. Untreated samples were blotted with non‐sensitive ECL; trametinib‐treated samples were blotted with sensitive ECL. Trametinib‐treated and non‐treated samples were compared to their sgCtrl. *n* = 2, *p < .05, **p < .01, ***p < .001, one‐way ANOVA followed by Tukey's test. (c) Cells stably expressing FLAG‐FBXO42 were immunoprecipitated with anti‐Flag. Bound proteins were eluted and analyzed by mass spectrometry. The identified proteins are listed. FLAG‐FBXO42ΔKelch and FLAG‐FBXO42ΔF‐box were used as control. (d,e) SK‐MEL‐147 and MZ‐MEL‐2 cell lines were treated with 100 nM trametinib for 24 hr. Cell lysates were analyzed by immunoblot with the indicated antibodies. pP38/P38 ratios were generated using Image Lab (Bio‐Rad). (f,g) Dose–response curves generated using SK‐MEL‐147 and MZ‐MEL‐2 cell lines, representing the delta between cells treated with trametinib (0.001–10 μM) and cells treated with the combination of trametinib (0.001–10 μM) and takinib (2.5 or 5 μM, respectively). Error bars, *SD*

MEK inhibitors have been shown to lead to insufficient MAPK pathway suppression and likely to pathway reactivation, a phenomenon known to be due to feedback reactivation (Merchant et al., 2017). To determine whether the resistance to trametinib in the *FBXO42* KO cells is due to activation of the MAPK pathway, we assessed RAS and ERK activation using lysates derived from the *FBXO42* KO cells in the presence or absence of trametinib and compared them to the control non‐targeting sgRNA cells. Indeed, we observed an increase in the expression levels of both RAS‐GTP and pERK in *FBXO42* KO samples treated and untreated with trametinib, compared to the control cells (Figure 5a,b and Figure S2), suggesting that KO of *FBXO42* in *NRAS*‐mutant melanoma cells leads to MAPK pathway activation.

### 3.4 Identification of novel FBXO42 binding partners

FBXO42 is an integral component of the SCF ubiquitin ligase complex. FBXO42 specifically associates with Skp1, Cul1, and Rbx1, the constituents of the SCF complex, an association which is dependent on the F‐box domain (Cardozo & Pagano, 2004; Petroski & Deshaies, 2005). The FBXO42 Kelch domain is responsible for the binding of an interactor protein regulated by SCF ubiquitination (Yan et al., 2015). In an effort to better understand the mechanistic role of FBXO42 in *NRAS*‐mutant melanoma, we performed a structure–function analysis of FBXO42 when devoid of its functional domains on the activation of the ERK pathway as a readout. Lysates from SK‐MEL‐147 cells stably expressing a F‐box domain‐deleted *FBXO42* mutant (FBXO42ΔF‐box) or Kelch domain‐deleted *FBXO42* mutant (FBXO42ΔKelch) showed an increase in pERK expression compared to the stable expression of WT FBXO42 (Figure S3). These results show that the FBXO42 functional domains F‐box and Kelch are necessary for the role of WT FBXO42 in inhibiting the MAPK pathway.

To gain insight into the molecular function of FBXO42, we investigated which proteins interact with it. Immunoprecipitation coupled with mass spectrometry analysis of SK‐MEL‐147 stably expressing either FBXO42 or control mutants FBXO42ΔKelch, FBXO42ΔFbox identified the presence of Skp1 and Cul1. These are components of the SCF complex, previously reported as FBXO42 interactors (Sun et al., 2009).

Interestingly, this analysis leads to the identification of a novel interaction with MAP3K7 also known as TAK1 and its regulators, TAB1, TAB2, and ITCH (Roh, Song, & Seki, 2014) (Figure 5c and Table S1). TAK1 activation is triggered by diverse stimuli, including pro‐inflammatory cytokines such as IL‐1 and TNF. TAK1 culminates in downstream activation of NF‐κB, P38, JNK, and ERK (Adhikari, Xu, & Chen, 2007; Ajibade et al., 2012). The binding to TAB1 and TAB2 proteins forms a complex required for TAK1 autophosphorylation‐induced activation. Furthermore, TAK1 is negatively regulated by the E3 ubiquitin ligase, ITCH (Roh et al., 2014; Shibuya et al., 1996).

We identified the indicated TAK1 binding partners only in WT FBXO42 and FBXO42ΔFbox samples but not in the FBXO42Δkelch sample, emphasizing that they might be potential interactors. These results suggest that FBXO42 has a role in the TAK1 signaling pathway.

### 3.5 *FBXO42* KO leads to TAK1 signaling activation

It has been shown that TAK1 phosphorylates and activated members of the mitogen‐activated protein kinase kinase (MKK) family, which, in turn, phosphorylate and activate JNK and P38 kinases (Adhikari et al., 2007; Ajibade et al., 2012). Indeed, an increase in pP38 expression, together with a decrease in p‐JNK, was identified in *FBXO42* KO samples compared to the control (Figure 5d,e and S4**)**. This is consistent with the ability of P38 MAPK to negatively regulate JNK (Gupta et al., 2015).

To further assess the involvement of the TAK1 signaling pathway in trametinib resistance in *NRAS* mutant *FBXO42* KO melanoma cells, we combined trametinib with takinib, the potent and selective TAK1 inhibitor (Totzke et al., 2017), and tested their inhibition of *FBXO42* KO cell growth. We found that they achieved greater efficacy than treatment with trametinib or takinib monotreatment (Figure 5f,g and Figure S5–7).

## 4 DISCUSSION

In recent years, the genetic landscape of melanoma has been extensively studied (Cancer Genome Atlas Network, 2015; Hodis et al., 2012; Krauthammer et al., 2012). On the basis of exome and genome sequencing studies, *BRAF* and *NRAS* were identified as the most commonly mutated genes in cutaneous melanoma patients (Krauthammer et al., 2012). As *NRAS* and *BRAF* activate the MAPK pathway, this led to the development of highly selective kinase inhibitors that target this pathway (Tsao, Chin, Garraway, & Fisher, 2012). However, acquired tumor resistance toward these targeted therapies is a significant therapeutic obstacle (Neel & Bivona, 2017). Thus, a better understanding of the pathways leading to such resistance is essential.

Here, a whole‐genome CRISPR‐Cas9 screen identified FBXO42 ubiquitin ligase involvement in resistance to trametinib treatment in the context of *NRAS*‐mutant melanoma. RNA sequencing analysis of *NRAS*‐mutant melanoma patient‐derived cell lines identified *FBXO42* to be differentially expressed between resistant and sensitive patients treated with MEK inhibitors.

Immunohistochemistry staining of these patient samples before and after MEKi treatment confirmed elevation in *FBXO42* expression in patients sensitive to MEKi. Complementary, *FBXO42* overexpression in MEKi‐resistant patient‐derived cell lines, decreases cell viability compared to control. This implies FBXO42 may be used as a biomarker for resistance in *NRAS*‐mutant melanoma.

*FBXO42* KO results in increased ERK activation, known to lead to proliferation and drug resistance in different types of cancer (McCubrey et al., 2007). Our findings indicate that FBXO42’s F‐Box and Kelch functional domains are both necessary for the inhibition of ERK activation.

Mass spectrometry analysis further showed that FBXO42 is involved in the TAK1 signaling pathway. TAK1 belongs to the MAP3K7 pathway activating P38, JNK, and NF‐κB signaling (Sakurai et al., 2002). It is well established that P38 signaling plays a central role in the regulation of cellular responses to stress, as well as the induction and progression of inflammation‐related diseases, inflammation‐induced cancer, and various cancers (Grossi, Peserico, Tezil, & Simone, 2014; Igea & Nebreda, 2015; Yin et al., 2016). Our findings add support to the attempt to try and inhibit this signaling pathway. Moreover, we demonstrate that combining the MEK inhibitor trametinib with the TAK1 inhibitor, takinib achieves far greater efficacy than treatment with trametinib alone in *NRAS*‐mutant melanoma cells.

The binding proteins TAB1‐3 enable phosphorylation and activation of TAK1 (Kanayama et al., 2004), whereas the E3 ubiquitin ligases RBCK1 and ITCH negatively regulate its activation. RBCK1 ubiquitinates TAB2 and TAB3, as ITCH ubiquitinates TAB1, resulting in proteasome‐dependent degradation (Hirata, Takahashi, Morishita, Noguchi, & Matsuzawa, 2017). We hypothesize, based on our findings, that FBXO42, which is too an E3 ligase, is a TAK1 negative regulator, possibly through TAB protein degradation, similarly to RBCK1 and ITCH regulation.

**Figure 6:**
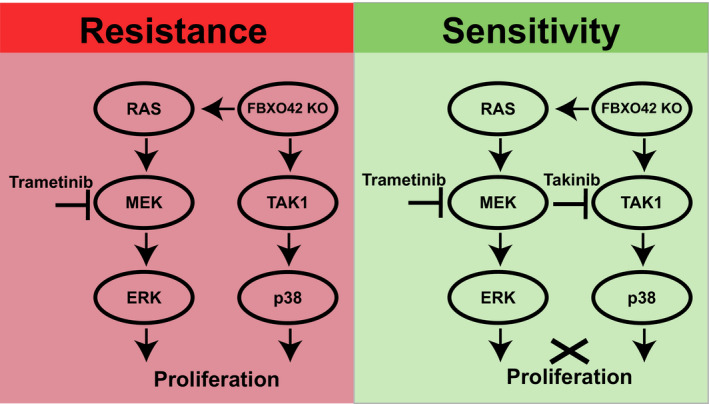
Graphical summary of signaling in *NRAS*‐mutant melanoma cells treated with trametinib. *FBXO42* KO in *NRAS*‐mutant melanoma cells leads trametinib resistance. This occurs by activation of the MAPK pathway together with TAK1 signaling through p38 activation. The combination of trametinib together with the TAK1 inhibitor, takinib, inhibits cellular proliferation

Put together, our findings reveal a novel mechanism leading to tumor resistance toward MEK inhibition in *NRAS*‐mutant melanoma (Figure 6). Namely, that FBXO42 has a role in trametinib resistance via the TAK1 signaling pathway, thus providing new opportunities for novel therapeutic options.

## CONFLICT OF INTEREST

The authors declare no ethical nor conflicts of interest.

## References

[pcmr12825-bib-0001] Adhikari, A. , Xu, M. , & Chen, Z. J. (2007). Ubiquitin‐mediated activation of TAK1 and IKK. Oncogene, 26(22), 3214–3226. 10.1038/sj.onc.1210413 17496917

[pcmr12825-bib-0002] Ajibade, A. A. , Wang, Q. , Cui, J. , Zou, J. , Xia, X. , Wang, M. , … Wang, R. F. (2012). TAK1 negatively regulates NF‐kappaB and p38 MAP kinase activation in Gr‐1+CD11b+ neutrophils. Immunity, 36(1), 43–54. 10.1016/j.immuni.2011.12.010 22226633 PMC3750978

[pcmr12825-bib-0003] Arafeh, R. , Qutob, N. , Emmanuel, R. , Keren‐Paz, A. , Madore, J. , Elkahloun, A. , … Samuels, Y. (2015). Recurrent inactivating RASA2 mutations in melanoma. Nature Genetics, 47(12), 1408–1410. 10.1038/ng.3427 26502337 PMC4954601

[pcmr12825-bib-0004] Cancer Genome Atlas Network (2015). Genomic classification of cutaneous melanoma. Cell, 161(7), 1681–1696. 10.1016/j.cell.2015.05.044 26091043 PMC4580370

[pcmr12825-bib-0005] Cardozo, T. , & Pagano, M. (2004). The SCF ubiquitin ligase: Insights into a molecular machine. Nature Reviews Molecular Cell Biology, 5(9), 739–751. 10.1038/nrm1471 15340381

[pcmr12825-bib-0006] Devitt, B. , Liu, W. , Salemi, R. , Wolfe, R. , Kelly, J. , Tzen, C. Y. , … McArthur, G. (2011). Clinical outcome and pathological features associated with NRAS mutation in cutaneous melanoma. Pigment Cell & Melanoma Research, 24(4), 666–672. 10.1111/j.1755-148X.2011.00873.x 21615881

[pcmr12825-bib-0007] Dobin, A. , Davis, C. A. , Schlesinger, F. , Drenkow, J. , Zaleski, C. , Jha, S. , … Gingeras, T. R. (2013). STAR: Ultrafast universal RNA‐seq aligner. Bioinformatics, 29(1), 15–21. 10.1093/bioinformatics/bts635 23104886 PMC3530905

[pcmr12825-bib-0008] Grossi, V. , Peserico, A. , Tezil, T. , & Simone, C. (2014). p38alpha MAPK pathway: A key factor in colorectal cancer therapy and chemoresistance. World Journal of Gastroenterology, 20(29), 9744–9758. 10.3748/wjg.v20.i29.9744 25110412 PMC4123363

[pcmr12825-bib-0009] Gupta, J. , Igea, A. , Papaioannou, M. , Lopez‐Casas, P. P. , Llonch, E. , Hidalgo, M. , … Nebreda, A. R. (2015). Pharmacological inhibition of p38 MAPK reduces tumor growth in patient‐derived xenografts from colon tumors. Oncotarget, 6(11), 8539–8551. 10.18632/oncotarget.3816 25890501 PMC4496165

[pcmr12825-bib-0010] Hirata, Y. , Takahashi, M. , Morishita, T. , Noguchi, T. , & Matsuzawa, A. (2017). Post‐translational modifications of the TAK1‐TAB complex. International Journal of Molecular Sciences, 18(1), 205– 10.3390/ijms18010205 PMC5297835 28106845

[pcmr12825-bib-0011] Hodis, E. , Watson, I. R. , Kryukov, G. V. , Arold, S. T. , Imielinski, M. , Theurillat, J. P. , … Chin, L. (2012). A landscape of driver mutations in melanoma. Cell, 150(2), 251–263. 10.1016/j.cell.2012.06.024 22817889 PMC3600117

[pcmr12825-bib-0012] Igea, A. , & Nebreda, A. R. (2015). The stress kinase p38alpha as a target for cancer therapy. Cancer Research, 75(19), 3997–4002. 10.1158/0008-5472.CAN-15-0173 26377941

[pcmr12825-bib-0013] Jakob, J. A. , Bassett, R. L. Jr , Ng, C. S. , Curry, J. L. , Joseph, R. W. , Alvarado, G. C. , … Davies, M. A. (2012). NRAS mutation status is an independent prognostic factor in metastatic melanoma. Cancer, 118(16), 4014–4023. 10.1002/cncr.26724 22180178 PMC3310961

[pcmr12825-bib-0014] Johnson, D. B. , & Puzanov, I. (2015). Treatment of NRAS‐mutant melanoma. Current Treatment Options in Oncology, 16(4), 15 10.1007/s11864-015-0330-z 25796376 PMC4830486

[pcmr12825-bib-0015] Kanayama, A. , Seth, R. B. , Sun, L. , Ea, C. K. , Hong, M. , Shaito, A. , … Chen, Z. J. (2004). TAB2 and TAB3 activate the NF‐kappaB pathway through binding to polyubiquitin chains. Molecular Cell, 15(4), 535–548. 10.1016/j.molcel.2004.08.008 15327770

[pcmr12825-bib-0016] Kim, D. W. , & Patel, S. P. (2014). Profile of selumetinib and its potential in the treatment of melanoma. OncoTargets and Therapy, 7, 1631–1639. 10.2147/OTT.S51596 25278770 PMC4179759

[pcmr12825-bib-0017] Krauthammer, M. , Kong, Y. , Ha, B. H. , Evans, P. , Bacchiocchi, A. , McCusker, J. P. , … Halaban, R. (2012). Exome sequencing identifies recurrent somatic RAC1 mutations in melanoma. Nature Genetics, 44(9), 1006–1014. 10.1038/ng.2359 22842228 PMC3432702

[pcmr12825-bib-0018] Law, C. W. , Chen, Y. , Shi, W. , & Smyth, G. K. (2014). voom: Precision weights unlock linear model analysis tools for RNA‐seq read counts. Genome Biology, 15(2), R29 10.1186/gb-2014-15-2-r29 24485249 PMC4053721

[pcmr12825-bib-0019] McCubrey, J. A. , Steelman, L. S. , Chappell, W. H. , Abrams, S. L. , Wong, E. W. , Chang, F. , … Franklin, R. A. (2007). Roles of the Raf/MEK/ERK pathway in cell growth, malignant transformation and drug resistance. Biochimica Et Biophysica Acta (BBA)‐Molecular Cell Research, 1773(8), 1263–1284. 10.1016/j.bbamcr.2006.10.001 17126425 PMC2696318

[pcmr12825-bib-0020] Merchant, M. , Moffat, J. , Schaefer, G. , Chan, J. , Wang, X. , Orr, C. , … Junttila, M. R. (2017). Combined MEK and ERK inhibition overcomes therapy‐mediated pathway reactivation in RAS mutant tumors. PLoS ONE, 12(10), e0185862 10.1371/journal.pone.0185862 28982154 PMC5628883

[pcmr12825-bib-0021] Munoz‐Couselo, E. , Adelantado, E. Z. , Ortiz, C. , Garcia, J. S. , & Perez‐Garcia, J. (2017). NRAS‐mutant melanoma: Current challenges and future prospect. OncoTargets and Therapy, 10, 3941–3947. 10.2147/OTT.S117121 28860801 PMC5558581

[pcmr12825-bib-0022] Neel, D. S. , & Bivona, T. G. (2017). Resistance is futile: Overcoming resistance to targeted therapies in lung adenocarcinoma. Npj Precision Oncology, 1(1), 3 10.1038/s41698-017-0007-0 29152593 PMC5687582

[pcmr12825-bib-0023] Petroski, M. D. , & Deshaies, R. J. (2005). Function and regulation of cullin‐RING ubiquitin ligases. Nature Reviews Molecular Cell Biology, 6(1), 9–20. 10.1038/nrm1547 15688063

[pcmr12825-bib-0024] Roh, Y. S. , Song, J. , & Seki, E. (2014). TAK1 regulates hepatic cell survival and carcinogenesis. Journal of Gastroenterology, 49(2), 185–194. 10.1007/s00535-013-0931-x 24443058 PMC3952073

[pcmr12825-bib-0025] Sakurai, H. , Nishi, A. , Sato, N. , Mizukami, J. , Miyoshi, H. , & Sugita, T. (2002). TAK1‐TAB1 fusion protein: A novel constitutively active mitogen‐activated protein kinase kinase kinase that stimulates AP‐1 and NF‐kappaB signaling pathways. Biochemical and Biophysical Research Communications, 297(5), 1277–1281. 12372426 10.1016/s0006-291x(02)02379-3

[pcmr12825-bib-0026] Santarpia, L. , Lippman, S. M. , & El‐Naggar, A. K. (2012). Targeting the MAPK‐RAS‐RAF signaling pathway in cancer therapy. Expert Opinion on Therapeutic Targets, 16(1), 103–119. 10.1517/14728222.2011.645805 22239440 PMC3457779

[pcmr12825-bib-0027] Shalem, O. , Sanjana, N. E. , Hartenian, E. , Shi, X. , Scott, D. A. , Mikkelson, T. , … Zhang, F. (2014). Genome‐scale CRISPR‐Cas9 knockout screening in human cells. Science, 343(6166), 84–87. 10.1126/science.1247005 24336571 PMC4089965

[pcmr12825-bib-0028] Shibuya, H. , Yamaguchi, K. , Shirakabe, K. , Tonegawa, A. , Gotoh, Y. , Ueno, N. , … Matsumoto, K. (1996). TAB1: An activator of the TAK1 MAPKKK in TGF‐beta signal transduction. Science, 272(5265), 1179–1182. 10.1126/science.272.5265.1179 8638164

[pcmr12825-bib-0029] Stephen, A. G. , Esposito, D. , Bagni, R. K. , & McCormick, F. (2014). Dragging ras back in the ring. Cancer Cell, 25(3), 272–281. 10.1016/j.ccr.2014.02.017 24651010

[pcmr12825-bib-0030] Sun, L. , Shi, L. , Li, W. , Yu, W. , Liang, J. , Zhang, H. , … Shang, Y. (2009). JFK, a Kelch domain‐containing F‐box protein, links the SCF complex to p53 regulation. Proceedings of the National Academy of Sciences, 106(25), 10195–10200. 10.1073/pnas.0901864106 PMC2700892 19509332

[pcmr12825-bib-0031] Thumar, J. , Shahbazian, D. , Aziz, S. A. , Jilaveanu, L. B. , & Kluger, H. M. (2014). MEK targeting in N‐RAS mutated metastatic melanoma. Molecular Cancer, 13(1), 45 10.1186/1476-4598-13-45 24588908 PMC3945937

[pcmr12825-bib-0032] Totzke, J. , Gurbani, D. , Raphemot, R. , Hughes, P. F. , Bodoor, K. , Carlson, D. A. , … Derbyshire, E. R. (2017). Takinib, a selective TAK1 inhibitor, broadens the therapeutic efficacy of TNF‐alpha inhibition for cancer and autoimmune disease. Cell Chemical Biology, 24(8), 1029–1039.e7, 10.1016/j.chembiol.2017.07.011 28820959 PMC5576570

[pcmr12825-bib-0033] Tsao, H. , Chin, L. , Garraway, L. A. , & Fisher, D. E. (2012). Melanoma: From mutations to medicine. Genes & Development, 26(11), 1131–1155. 10.1101/gad.191999.112 22661227 PMC3371404

[pcmr12825-bib-0034] Yan, R. , He, L. , Li, Z. , Han, X. , Liang, J. , Si, W. , … Shang, Y. (2015). SCF(JFK) is a bona fide E3 ligase for ING4 and a potent promoter of the angiogenesis and metastasis of breast cancer. Genes & Development, 29(6), 672–685. 10.1101/gad.254292.114 25792601 PMC4378198

[pcmr12825-bib-0035] Yin, N. , Qi, X. , Tsai, S. , Lu, Y. , Basir, Z. , Oshima, K. , … Chen, G. (2016). p38gamma MAPK is required for inflammation‐associated colon tumorigenesis. Oncogene, 35(8), 1039–1048. 10.1038/onc.2015.158 25961922

