# Supplementary material for: A genome‐wide CRISPR screen identifies FBXO42 involvement in resistance toward MEK inhibition in NRAS‐mutant melanoma
Source: Pigment Cell Melanoma Res. 2019 Oct 10;33(2):334–44. doi: 10.1111/pcmr.12825 (PMC7383499; doi:10.1111/pcmr.12825)
Supplement: Supplementary file 1 [file PCMR-33-334-s001.docx]

**Supplementary information**

**A genome-wide CRISPR screen identifies *FBXO42* involvement in resistance towards MEK inhibition in *NRAS* mutant melanoma**

Adi Nagler^1^, David W. Vredevoogd^2^, Michal Alon**^1^**, Phil F. Cheng^3^, Sophie Trabish^1^, Shelly Kalaora**^1^**, Rand Arafeh**^1^**,Victoria Goldin^1^, Mitchell P. Levesque^3^, Daniel S. Peeper^2^* and Yardena Samuels^1^*

^1^ Molecular Cell Biology Department, Weizmann Institute of Science, Rehovot, Israel.

^2^ Division of Molecular Oncology and Immunology, The Netherlands Cancer Institute, Plesmanlaan 121, 1066 CX Amsterdam, The Netherlands.

^3^ Department of Dermatology, University of Zurich Hospital, Zurich, Switzerland.

To whom correspondence should be addressed:

Email: [Yardena.samuels@weizmann.ac.il](https://xmail.weizmann.ac.il/owa/redir.aspx?C=1voMRn0xLa5UBYtCl8oZ9wxJ33JWHPQ2pbCduyLFLtLSz9hkZUnWCA..&URL=mailto%3aYardena.samuels%40weizmann.ac.il)

Email: [d.peeper@nki.nl](mailto:d.peeper@nki.nl)

**Supplemental Figure 1**


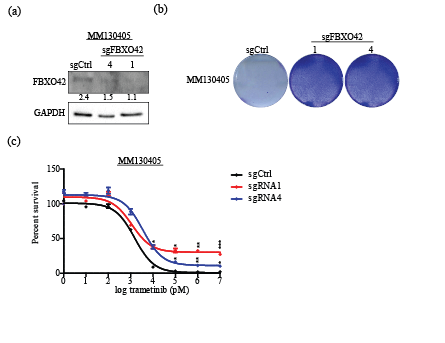


**Supplemental Figure 1. FBXO42 KO leads to trametinib resistance in MM130405 cells.** a) Immunoblot analysis of *FBXO42* gene perturbation efficiency in MM130405 patient derived *NRAS* mutant melanoma cells. Ratios of FBXO42 to GAPDH levels were generated using Image lab (BioRad). b) MM130405 cells were treated with 10 nM trametinib and stained with crystal violet 10 days later. c) Dose response curves generated using MM130405 cells treated with trametinib

(1 pM – 10 μM) for 72 hr before assessing viability with the Cell Titer-Glo Luminescent Cell viability assay. n=3, *p<0.05, **p<0.01, ***p<0.001, two-way ANOVA followed by Bonferoni’s post-hoc test. The relative cell number post-trametinib treatment is plotted as percent survival versus log trametinib concentration in pM. Error bars, s.d.

**Supplemental Figure 2**


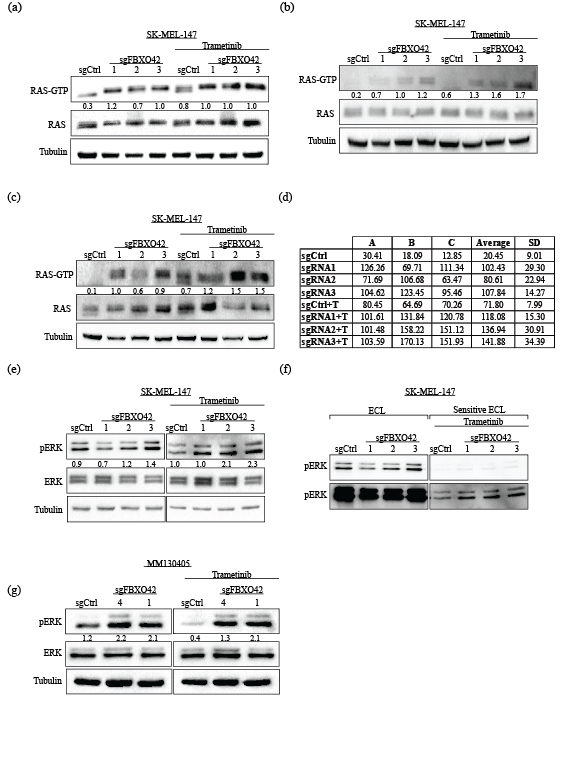


**Supplemental Figure 2. *FBXO42* KO activates the MAPK pathway.** a-c) Western blot of RAS-GTP levels in SK-MEL-147 cell line after treatment with 100 nM trametinib for 24 hr. RAS-GTP levels were assessed by a RAS pulldown assay after treatment. Blots presented were used for quantification in Figure 5A. d) Quantification values of western blots A-C used for graph 5A. e) SK-MEL-147 cells were treated with 100 nM trametinib for 24 hr. Cell lysates were analyzed by western blot with the indicated antibodies. Representative blot used for quantification in Figure 5B. f) Original blots of Figure E, untreated samples were blotted with non sensitive ECL; trametinib-treated samples were blotted with sensitive ECL. g) MM130405 cells were treated with 10 nM trametinib for 24 hr. Cell lysates were analyzed by western blot with the indicated antibodies. Western blot for samples treated with trametinib (Right) was developed with sensitive ECL. Ratios were generated using Image lab (BioRad).

**Supplemental Figure 3**


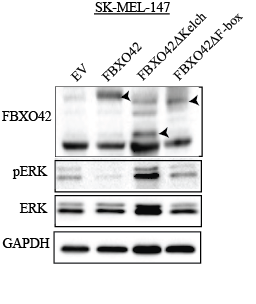


**Supplemental Figure 3. Deletion of *FBXO42* functional domains effect ERK regulation.** Lysates from SK-MEL-147 cells expressing FLAG-FBXO42 or FLAG-FBXO42ΔKelch, or FLAG-FBXO42ΔF-box were immunoblotted with the indicated antibodies. Arrows indicating FBXO42 or FBXO42 mutant expression.


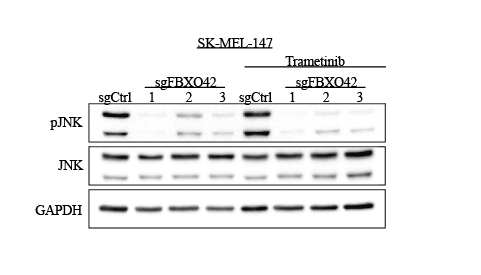
 **Supplemental Figure 4**

**Supplemental Figure 4. pJNK decrease in SK-MEL-147 *FBXO42* KO cells.**

SK-MEL-147 cells treated with 100 nM trametinib for 24 hr. Cell lysates were analyzed by immunoblot with the indicated antibodies.


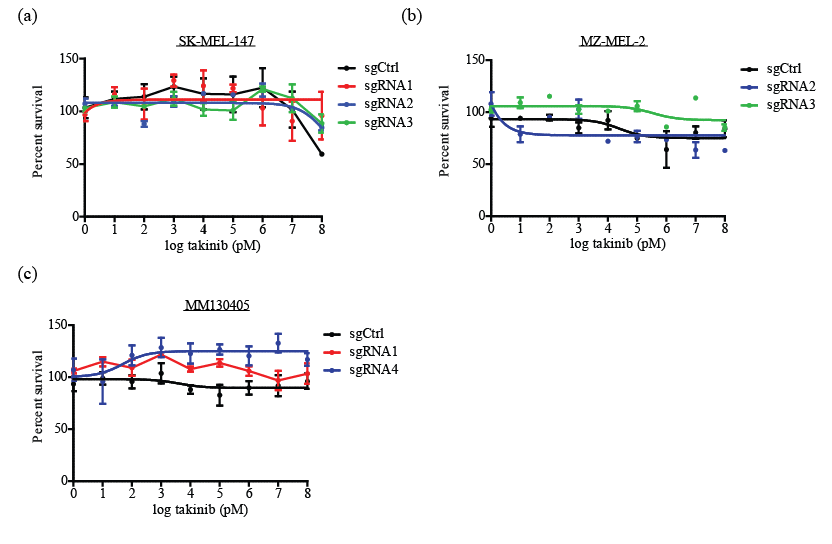
**Supplemental Figure 5**

**Supplemental Figure 5. The effect of TAK1 inhibitor, takinib, on *NRAS* mutant melanoma cell lines.** a-c) Dose response curves generated using SK-MEL-147, MZ-MEL-2 and MM130405 cell lines treated with takinib (1 pM – 100 μM) for 72 hr before assessing viability with the Cell Titer-Glo Luminescent Cell viability assay (n=3). The relative cell number post-takinib treatment is plotted as percent survival versus log takinib concentration in pM. Error bars, s.d.

**Supplemental Figure 6**


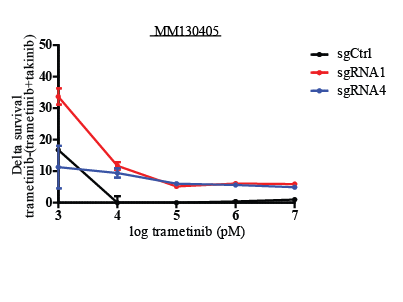


**Supplemental Figure 6. Combination of MEK and TAK1 inhibition is more efficient than monotreatment with MEKi in MM130405 cells.** Dose response curves generated using MM130405 cell line, representing the delta between cells treated with trametinib (0.001-10 μM) to cells treated with the combination of trametinib (0.001-10 μM) and takinib (5 μM). Error bars, s.d.

**Supplemental Figure 7**


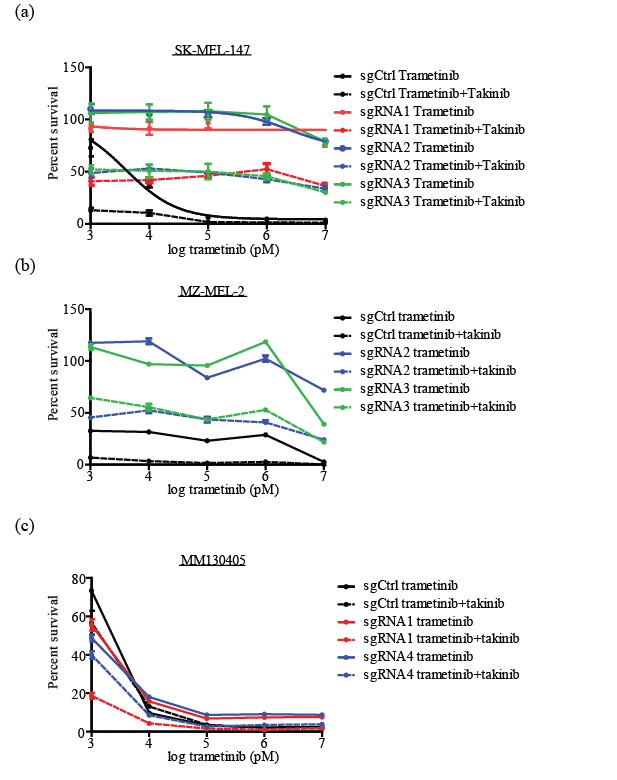


**Supplemental Figure 7. Combination of MEK and TAK1 inhibition is more efficient than monotreatment with MEKi.** a) Dose resopnse cerves representing the relative cell number post treatment is plotted as percent survival versus log trametinib concentration. SK-MEL-147 cells treated with trametinib (0.01-100 μM) or the combination of trametinib (0.01-100 μM) and takinib (2.5 μM). b,c) Dose resopnse cerves of MZ-MEL-2 and MM130405 treated with trametinib (0.01-100 μM) or the combination of trametinib (0.01-100 μM) and takinib (5 μM) . Error bars,s.d.

**Supplemental Figure 8**


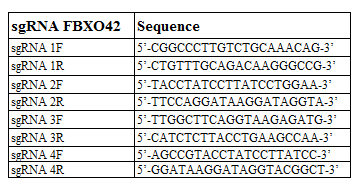


**Supplemental Figure 8.** **sgRNA sequences used for generation of *FBXO42* KO**.

All sgRNA sequences are from the GeCKO v2 library.
